# The effect of capitalization on financial return in periodic growth

**DOI:** 10.1016/j.heliyon.2019.e02728

**Published:** 2019-11-01

**Authors:** Petri P. Kärenlampi

**Affiliations:** Lehtoi Research, Lehtoi, Finland

**Keywords:** Economics, Capital return, Appreciation, Real estate, Probability density function

## Abstract

A capital return rate function for growth processes is introduced and applied to financial considerations in periodically growing multiannual plants. The capital return rate function is composed of a momentary capital return function, a probability density function in the time domain, and their integration over time or age. It is shown that the expected value of capital return rate within a single stand equals momentary capital return rate within an estate, integrated over an even distribution of stand ages. We distribute the capitalization to operative and non-operative capitalization. In the case of a low non-operative capitalization, financially sound operations favor relatively small amount of operative capital. In the case of a high, but constant non-operative capitalization, optimal practices correspond to those resulting in maximum sustainable yield. Appreciating non-operative capitalization favors small operative capitalization. Optimal rotation and operative capitalization are weak functions of increasing level of non-operative capitalization, even if they are strong functions of its increment rate. It is argued that large but non-appreciating non-operative capitalization, favoring practices corresponding to maximum sustainable yield, would not appear frequently. In summary, it is found that appreciation of non-operative capitalization dominates financially sustainable management practices.

## 1 Introduction

Businesses should be sustainable. In businesses involved in growing multiannual plants, sustainability may refer to maintenance of growing stock, maintenance of growth, or maintenance of productive area (Kuusela, 1961; Posavec et al., 2012). Another view is that maintenance is not necessarily enough, there possibly should be a progression in the amount of growing stock, growth, or possibly productive area (Kuusela, 1961). Financial considerations have been incorporated to sustainability criteria. Most commonly, a discounting interest rate is applied in order to compute the present value of future incomes and expenses (Faustmann, 1849; Pearse, 1967; Samuelson, 1976; Yin and Newman, 1995; Deegen et al., 2011; Campbell, 1999; Nyyssönen, 1999; Tahvonen, 2016; Gong and Löfgren, 2016; Abdallah and Lasserre, 2017). The discounting interest may vary over time (Price, 2011, 2017a; Buongiorno and Zhou, 2011; Brazee, 2017). It has been stated that uncertainty induces declining discount rates along with time (Groom et al., 2005; Price, 2011, 2017b; Hepburn and Koundouri, 2007); however it can be shown that there is an opposite effect on prolongation interest. Risk of destructive events has been considered as a premium to discount interest (Loisel, 2011; Hyytiäinen and Haight, 2010). Evolution of prices, as well as fluctuations in growth and prices may be added (Buongiorno and Zhou, 2011; Yin and Newman, 1997). Taxation does contribute, as well as personal financies (Koskela and Alvarez, 2004; Tahvonen and Salo, 1999; Tahvonen et al., 2001). A Hamiltonian formulation is available (Termansen, 2007).

It has recently been shown that maximization of net present value of future net proceeds may lead to financially devastating consequences (Kärenlampi, 2019a). An obvious reason for this is that a discounting interest rate is taken externally, were as optimization of capital return rate should be based on the features of any production process. Such results have been gained in the case of stationary rotation forestry (Kärenlampi, 2019a). Stationarity here means that stand ages are evenly distributed, and the even stand age distribution is retained by regular regeneration. Stationarity, in the financial sense, also requires that prices and expenses, including non-operative valuations like an eventual bare land value, do not evolve in real terms. It has been shown that maximizing an internal rate of return (IRR: Boulding, 1955; Newman, 1988) yields only slightly biased results in stationary forestry (Kärenlampi, 2019a). Maximization of the net present value of future proceeds becomes similar to the IRR, provided the discounting interest rate is calibrated to yield a realistic bare land value (Kärenlampi, 2019a).

Another interesting development regards financial performance of continuous-cover forestry (Kärenlampi, 2018). Frequent diameter-limit harvestings, in the absence of any regeneration cycles, result as essentially stationary operation. However, such a system may be financially nonstationary, provided prices or expenses evolve (Kärenlampi, 2018). A low, stationary non-operative capitalization appears to favor financially a small volume of standing trees. A large non-operative capitalization requires a large amount of standing trees, corresponding to a large cutting limit diameter (Kärenlampi, 2018). Finally, a significant appreciation rate on non-operative capitalization requires a small volume of standing trees, corresponding to a low cutting limit diameter (Kärenlampi, 2018).

In this paper, we investigate capital return in the business of growing multiannual plants in a periodic manner. We do not apply any financial stationarity criterion. Instead, we investigate the effects of eventually evolving capitalization. A momentary return rate of capital is defined, as well as its probability density function. Then, the capital return is integrated over time on the one hand, and over age on the other hand (cf. Kärenlampi, 2019b).

The effect of non-operative capitalization on the financial sustainability is investigated within the frameworks of two practical forestry examples (Gong and Löfgren, 2016). The effect of stationary non-operative capitalization on the capital return is investigated, as well as consequent optimal rotation. Then, the effect of appreciating non-operative capitalization on operative and total capital return is studied. Thirdly, long-term solutions are developed, covering many rotation cycles. The latter formulations are solved under the boundary condition of high non-operative capitalization. A few cases of intermediate non-operative capitalization are discussed. The methods introduced could be used to any growth process, provided a yield function can be approximated.

## 2 Model

### 2.1 State-space capital return model

Let us first write a momentary rate of capital return, in terms of capitalization *K* and time *t*:

(1) $$r\left(t\right)=\frac{d\kappa }{K\left(t\right)dt}$$

By definition, the expected value of the capital return rate is

(2) 〈r〉=∫rp(r)dr

where *p* refers to a probability density function. In the time domain, Eq. (2) becomes

(3) $$\langle r\rangle =\int_{0}^{\tau }rp\left(r\right)\frac{dr}{dt}dt$$

where τ is rotation time, and

(4) $$p\left(r,t\right)=p\left(r\right)\frac{dr}{dt}$$

is the probability density function of capital return rate in the time domain. On the other hand, the expected value of the capital return rate is

(5) $$\langle r\rangle =\frac{\langle \frac{d\kappa }{dt}\rangle }{\langle K\rangle }=\frac{\int_{0}^{\tau }\frac{d\kappa }{dt}dt}{\int_{0}^{\tau }Kdt}=\frac{\int_{0}^{\tau }rKdt}{\int_{0}^{\tau }Kdt}$$

where the numerator is written in terms of the partition function (or state sum) of the change rate of capitalization, and the denominator is the partition function of the capitalization (Kärenlampi, 2019b). Comparing Eqs. (3), (4) and (5), we find that the probability density function of capital return rate in the time domain is

(6) $$p\left(r,t\right)=\frac{K\left(t\right)}{\int_{0}^{\tau }Kdt}$$

In Eqs. (1) and (5), the difference between κ in the numerator and *K* in the denominator relates to eventual operative investment. The capitalization *K* is immediately affected by any eventual operative investment (or withdraval), and then consequently becomes affected by amortizations. The net return rate $\frac{d\kappa }{dt}$ in the numerator possibly is indirectly affected by investments through increased growth, *etc.,* but apart from that, considers investments in terms of amortizations only. Even amortizations of operative investments throughout the applied (or remaining) rotation age are applied.

The Equations above were written in the time domain. That is not the only possibility. Let us convert some of them into the domain of stand age. Firstly, an expected value of capitalization is, by definition,

(7) $$\langle K\rangle =\int_{0}^{\infty }p\left(K\right)KdK$$

where p(K) is the probability density function of capitalization K. By change of variables we get

(8) $$\langle K\rangle =\int_{0}^{\tau }p\left(K\right)K\frac{dK}{da}da=\int_{0}^{\tau }p\left(a\right)K\left(a,t\right)da$$

where *a* is stand age, and *τ* is rotation age. The expected value of the increment rate of capitalization is

(9) $$\langle \frac{d\kappa }{dt}\rangle =\int_{0}^{\tau }p\left(a\right)\frac{d\kappa \left(a,t\right)}{dt}da$$

Correspondingly, the momentary expected rate of relative capital return is

(10) $$\langle r\left(t\right)\rangle =\frac{\langle \frac{d\kappa }{dt}\rangle }{\langle K\rangle }=\frac{\int_{0}^{\tau }p\left(a\right)\frac{d\kappa \left(a,t\right)}{dt}da}{\int_{0}^{\tau }p\left(a\right)K\left(a,t\right)da}=\frac{\int_{0}^{\tau }p\left(a\right)\kappa \left(a,t\right)r\left(a,t\right)da}{\int_{0}^{\tau }p\left(a\right)K\left(a,t\right)da}$$

Now, Eq. (10) equals Eq. (5), provided there is an even distribution of stand ages (p(a) is constant). Even if the outcome is the same, the derivations are rather different; Eq. (10) discusses an estate with a variety of stand ages, whereas function (5) may well be applied to any single stand. Interestingly, Eq. (10) indicates that the capitalization, as well as the capital return rate, may be functions of two separate variables, time and stand age. It is further worth noting that even apart from a “normal forest” boundary condition (Leslie, 1966) the constancy of the probability density of stand age within the integration range is valid when discussing one single stand.

Let us now develop Eq. (5) further by introducing an operative and a non-operative component of capitalization. Operative capitalization component appreciates through growth. Non-operative capitalization may be due to excess demand of real estate in comparison to supply, recreational values, speculation for future real estate development, etc. We include any bare land value in the non-operative capitalization. The operative capitalization is denoted as O(t), non-operative capitalization as U(t), and rewrite Eq. (5) as

(11) $$\langle r\rangle =\frac{1}{\int_{0}^{\tau }\left(O+U\right)dt}\int_{0}^{\tau }\frac{d\left(\Omega +U\right)}{dt}dt=\frac{\Delta \Omega \left(\tau \right)+\Delta U\left(\tau \right)}{\tau \left[\langle O\rangle +\langle U\rangle \right]}$$

In Eq. (11), the difference between Ω in the numerator and *O* in the denominator again relates to eventual operative investment. The capitalization *O* is immediately affected by any eventual operative investment, and then consequently becomes reduced by amortizations. The net return rate $\frac{d\Omega }{dt}$ in the numerator considers investments in terms of amortizations only. Correspondingly, the accumulated net yield ΔΩ(τ) may differ from net change in operative capitalization ΔO(τ) in the occurrence of withdrawals (harvesting etc.).

We find from Eq. (11) that in the case the operative capitalization is much higher than the non-operative capitalization, the role of the latter vanishes. If non-operative capitalization is much higher than operative capitalization, the role of the operative capitalization vanishes. In case the non-operative capitalization is large but constant, the highest operative return would simply correspond to the greatest average yieldΔΩ(τ)/τ. The situation is more complicated if there is a nonvanishing time change rate of the non-operative capitalization dU/dt.

## 3 Results

### 3.1 A volumetric yield example

As a practical forestry example, we consider two recently introduced (Gong and Löfgren, 2016) yield functions, applicable to average pine stands in Northern Sweden. A volumetric growth function is

(12) $$V\left(t\right)=580.14*{\left(1-6.3582^{-t/95}\right)}^{2.8967}$$

The first application introduced by Gong and Löfgren (2016) assumes a stumpage price of 250 SEK/m^3^, and an initial investment of 6000 SEK/ha. The maximum sustained yield rotation being 95 years, a 3% discount interest would yield an optimal rotation age of 52 years (Gong and Löfgren, 2016). We now apply Eq. (11) for this case.

The non-operative capitalization U should be parametrized somehow. We choose to fix the ratio UO at the time instant of 100 years. We first establish a treatment where the non-operative capitalization is constant in time. In other words, dU/dt=0. Then we apply nonzero change rates for the non-operative capitalization, using a few alternative appreciation rates. We first discuss the operative return rate of capital, and then the total return rate of capital.

**Fig. 1:**
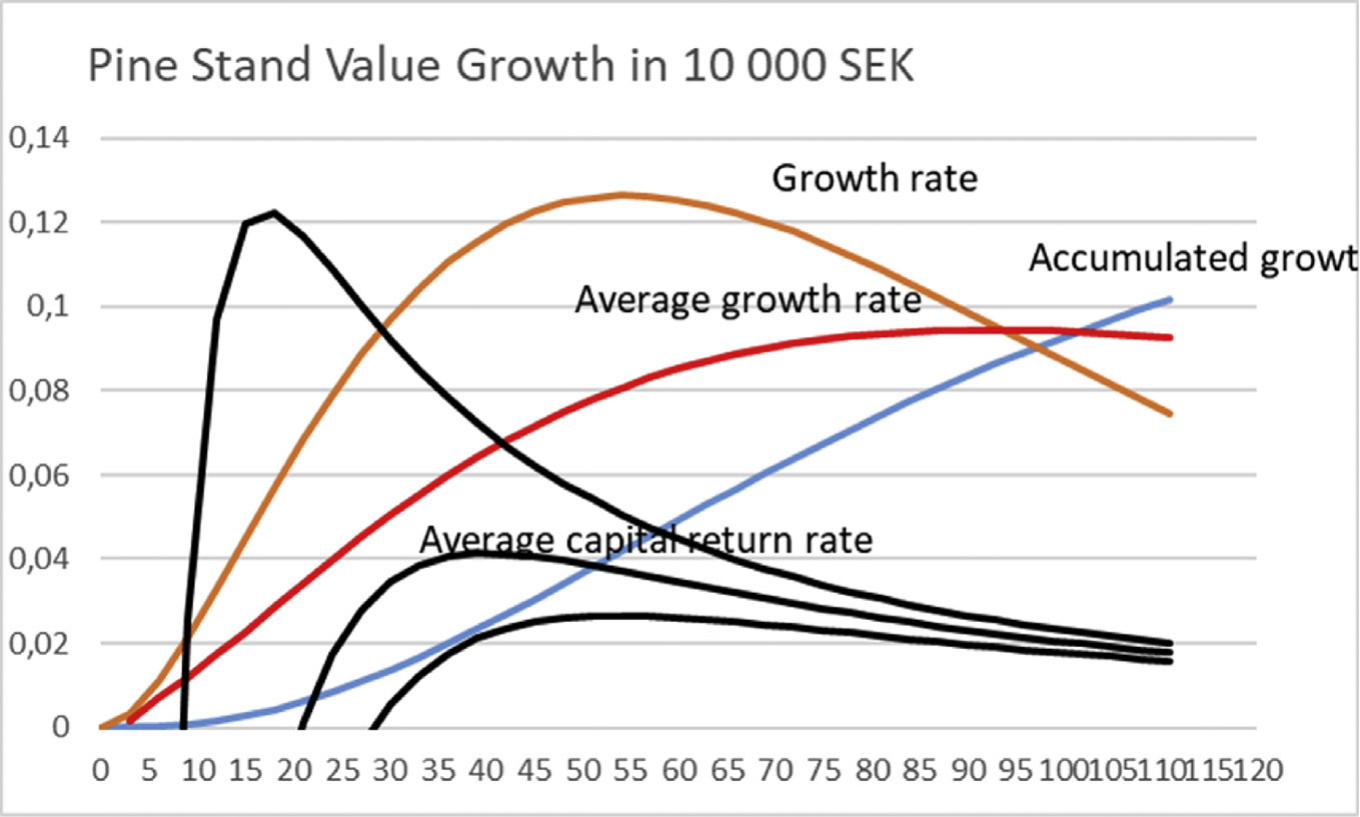
Pine stand value growth according to a North-Swedish growth function (12) (Gong and Löfgren, 2016). Solid black lines correspond to average financial return rate according to Eq. (3) for three different levels of initial investment, with 〈U〉〈O〉=0. Value growth is given in units of 10 000 SEK, whereas the accumulated growth in millions per hectare.

In Fig. 1, the non-operative capitalization is set as zero. In other words, [UO]100=0. Fig. 1 shows the operative capital return of 4.18% at rotation time 40 years, provided the initial investment is SEK 6000, as approximated by Gong and Löfgren (2016).

If the growth could be initiated with a tiny investment of 600 SEK, operative capital return of 13.1% would be achieved at 17 years of rotation (Fig. 1). In case the initial investment would have to be doubled to 12 000 SEK, maximum capital return of 2.67% would be achieved at 54 years of rotation.

**Fig. 2:**
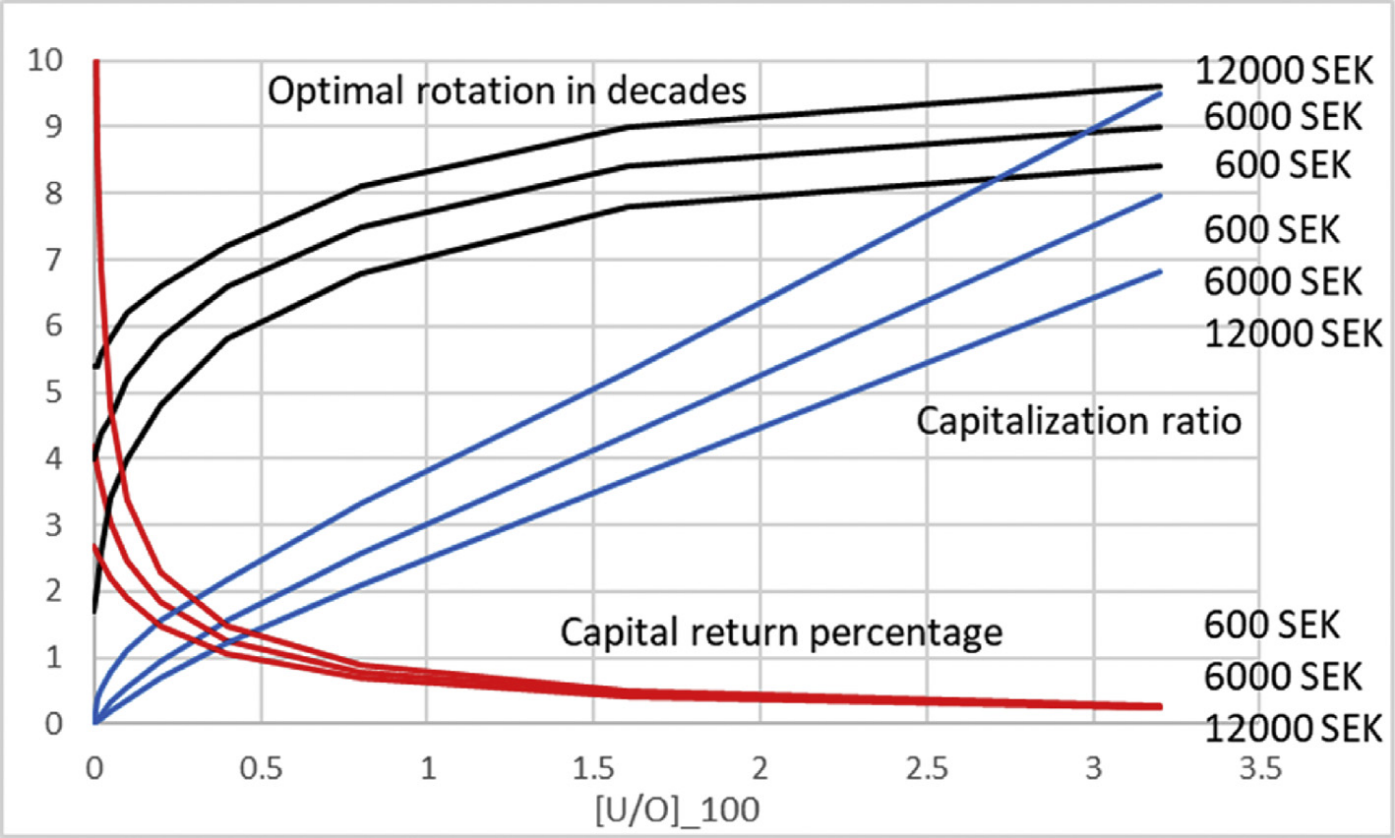
Optimal rotation time, average capitalization ratio 〈U〉〈O〉, and capital return percentage as a function of [UO]100 for three different levels of initial investment for dU/dt=0.

In Fig. 2 we introduce a nonzero non-operative capitalization, but retain dU/dt=0. We plot the optimal rotation time, average capitalization ratio 〈U〉〈O〉 at optimal rotation, as well as the capital return percentage as a function of fixed [UO]100. [UO]100=0 on the left naturally corresponds to the situation illustrated in Fig. 1. Then, as a function of increasing non-operative capitalization, the optimal rotation time evolves towards 95 years, corresponding to greatest possible average net yield rate ΔΩ(τ)/τ, or equivalently the maximum sustainable yield (MSY). Simultaneously, the operative capital return rate becomes reduced, and the average capitalization ratio at optimum rotation time 〈U〉〈O〉 increases. Results are plotted for three different values of initial investment, similarly to Fig. 1, and they differ the most at small non-operative capitalization.

**Fig. 3:**
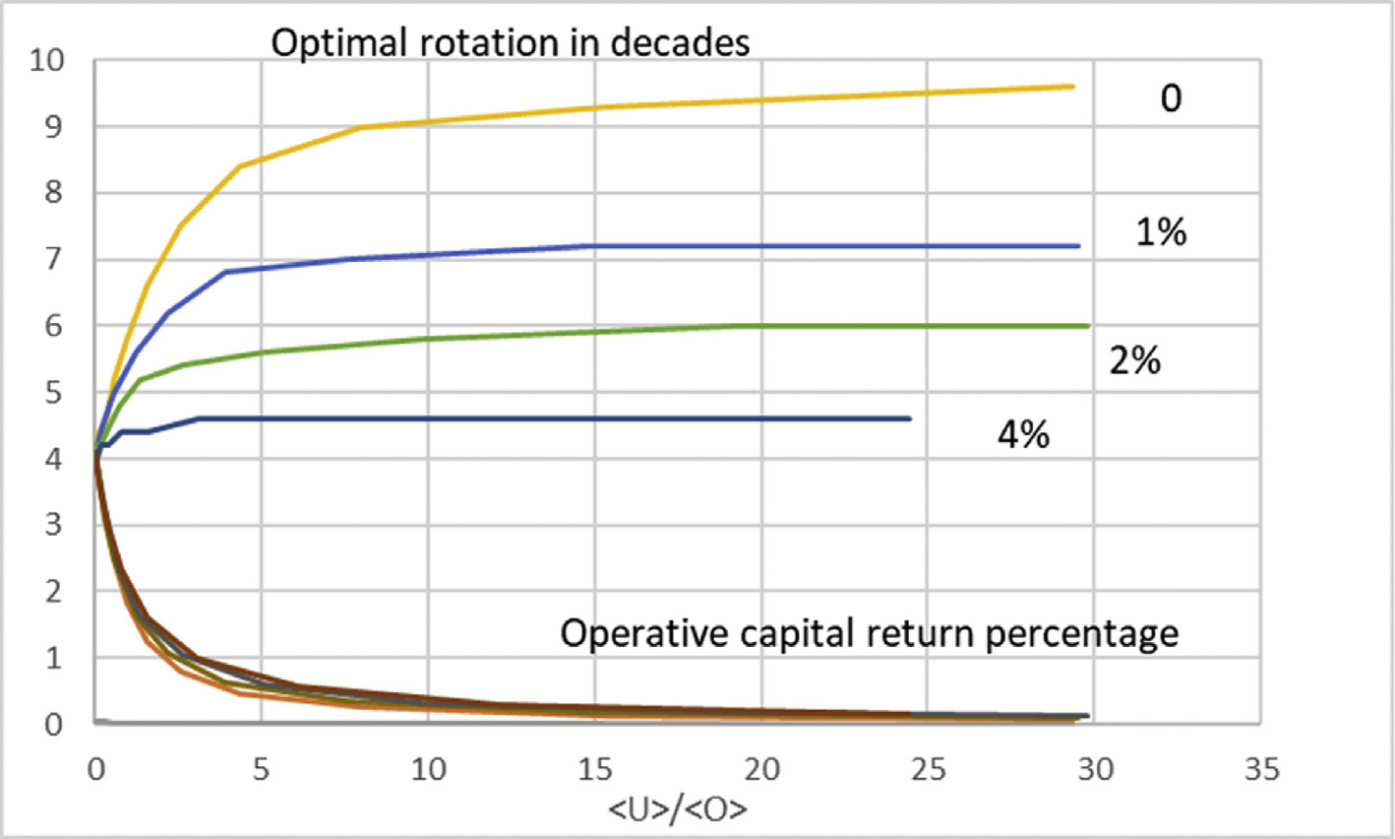
Optimal rotation time and operative capital return percentage as a function of average capitalization ratio 〈U〉〈O〉 at optimum rotation, for four different levels of annual appreciation of non-operative capitalization.

In Fig. 3, we retain a variety of values of non-operative capitalization, but introduce a nonzero dU/dt. There is only one value of initial investment, 6000 SEK/ha. We arrange the data according to average capitalization 〈U〉〈O〉 at the instant of maximum operative capital return $\frac{\Delta \Omega \left(\tau \right)}{\tau \left[\langle O\rangle +\langle U\rangle \right]}$ (cf. Eq. (11)). Interestingly, the maximal operative capital return is almost independent on the appreciation rate of the non-operative capitalization. Still more interestingly, the optimal rotation time is rather sensitive to the appreciation rate. Perhaps most interestingly, the optimal rotation age is a rather weak function of the level of non-operative capitalization; in the case of 4% appreciation, increasing non-operative capitalization does not increase the optimal rotation time beyond 46 years.

**Fig. 4:**
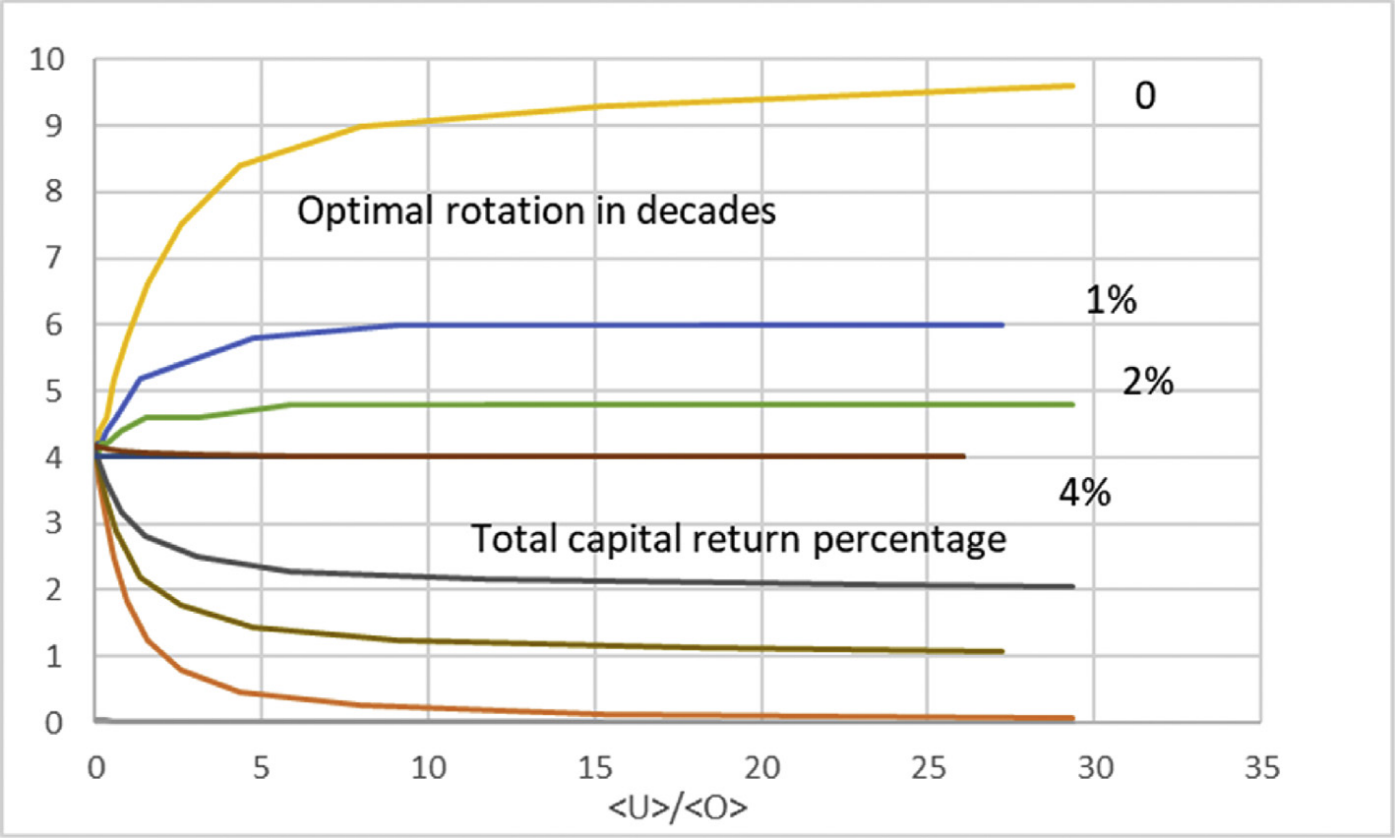
Optimal rotation time and total capital return percentage as a function of average capitalization ratio 〈U〉〈O〉 at optimum rotation, for four different levels of annual appreciation of non-operative capitalization.

In Fig. 4 we discuss total return of capital, instead of the operative return. We again arrange the data according to average capitalization 〈U〉〈O〉, but now at the instant of maximum total capital return *u* (cf. Eq. (11)). It is found that the maximal total capital return approaches the appreciation rate of the non-operative capitalization along with increasing non-operative capitalization. More interestingly, the non-operative appreciation rate strongly contributes to the effect of non-operative capitalization on the optimal rotation time. At zero appreciation, the optimal rotation time approaches the Maximum Sustainable Yield rotation along with non-operative capitalization, as already recognized from Eqs. (1), (2) and (3) and from Figs. 2 and 3. However in the case of appreciating non-operative capitalization this does not happen. As a function of increasing non-operative capitalization, the optimal rotation time approaches a constant that rather significantly differs from the MSY-rotation. Most interestingly, in the case of 4% annual non-operative appreciation, the optimal rotation time never increases from 40 years, which is the optimum at zero non-operative capitalization.

**Fig. 5:**
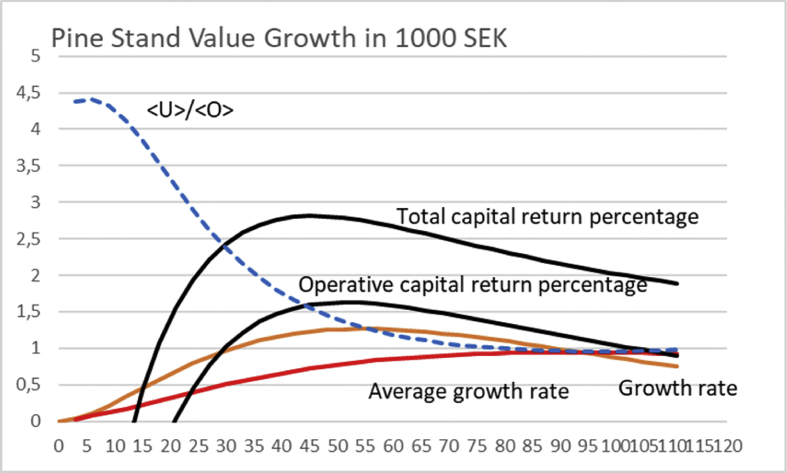
Total and operative capital return percentages as a function of rotation time for Pine stand growth function (12). The Figure also displays average capitalization ratio 〈U〉〈O〉. Non-operative capital appreciation rate is 2%, and [UO]100=1.

Deeper understanding of the results shown in Figs. 3 and 4 probably requires discussion of capital return as a function of rotation time in the presence on non-operative capitalization. We show the operative and the total capital return as a function of rotation time in Fig. 5, where [UO]100=1, and non-operative capital appreciation rate is 2%. It is found that the maximum total capital return rate is found at rotation time 46 years, where the average capitalization ratio is 1.53. The maximum operative capital return rate is found at rotation time 52 years, where the average capitalization ratio is 1.365. Beyond 100 years of rotation, the total capital return rate is below the non-operative capital appreciation rate.

**Fig. 6:**
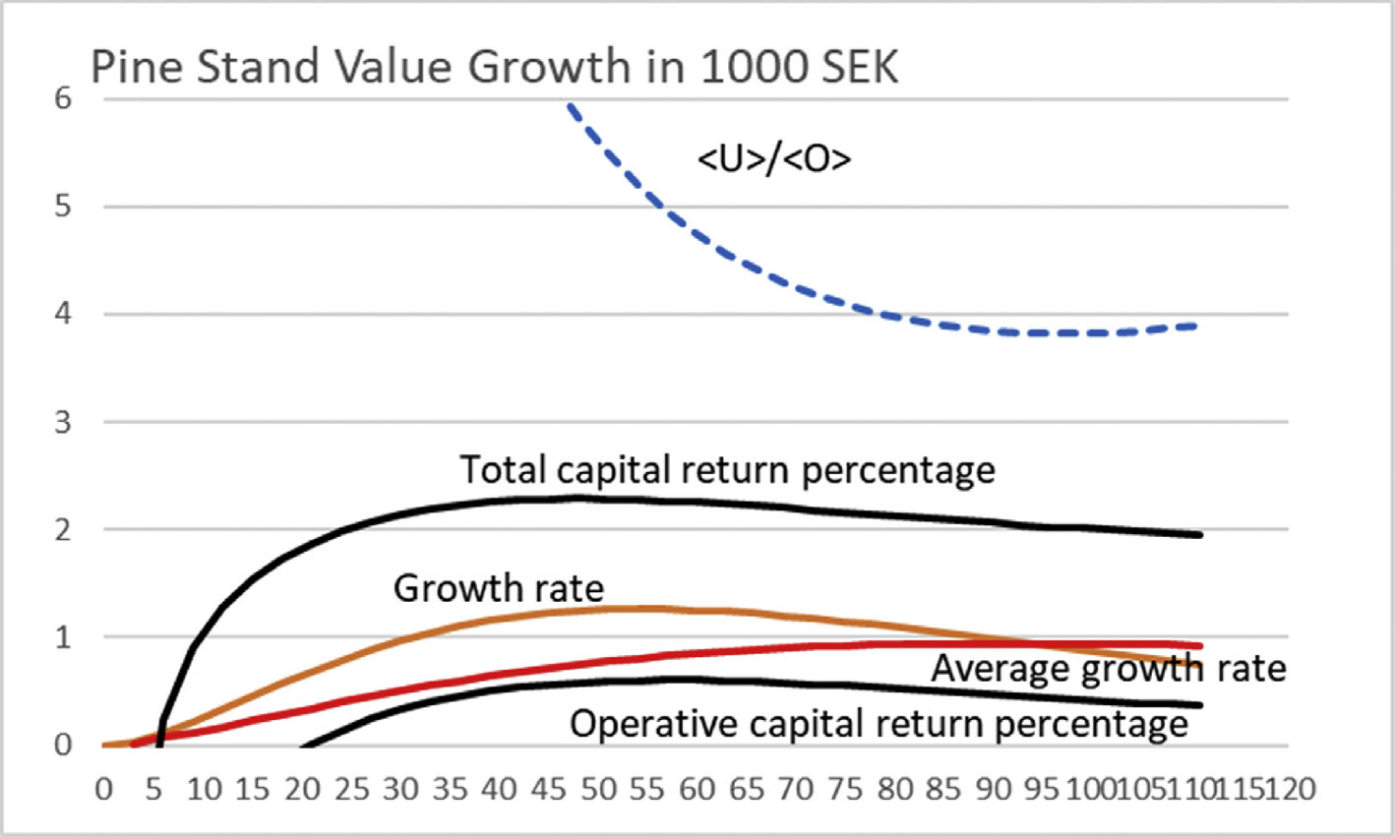
Total and operative capital return percentages as a function of rotation time for Pine stand growth function (12). The Figure also displays average capitalization ratio 〈U〉〈O〉. Non-operative capital appreciation rate is 2%, and [UO]100=4.

One can readily recognize that the situation depicted in Fig. 5 is not stationary in the long term. In case the non-operative capitalization keeps appreciating, the boundary condition [UO]100 evolves. With 2% appreciation rate of the non-operative capitalization, after 35 years [UO]100=2. After another 35 years, [UO]100=4. Fig. 6 shows the capital return rates, as well as the capitalization ratio, for the latter value of the boundary condition. We find that the maximum total capital return rate is found at rotation time 48 years, where the average capitalization ratio is 5.86. The maximum operative capital return rate is found at rotation time 56 years, where the average capitalization ratio is 5.06. Beyond 100 years of rotation, the total capital return rate is below the non-operative capital appreciation rate. In accordance with Figs. 3 and 4, the optimal rotation time somewhat increases along with non-operative capitalization, but the increment ceases along with further non-operative capitalization. A comparison of Figs. 5 and 6 shows that the capital return curve as a function of rotation time becomes flatter along with increasing non-operative capitalization. However there is a distinct increment of the capital return at low rotation times, related to amortization of the initial operative investment.

It is worth noting that at the 2% appreciation rate the non-operative capitalization, the average capitalization ratio 〈U〉〈O〉 predominantly decreases along with increasing age, due to increasing operative capitalization *O* (Figs. 5 and 6). This is not necessarily the case if the appreciation rate of the non-operative capitalization is greater than 2%.

### 3.2 A value growth example

Eq. (12), as well as Figs. 1, 2, 3, 4, 5, and 6, assumes the real-valued volumetric stumpage price to be constant. In other words, the value growth corresponds to volumetric growth, multiplied by a constant. That may be an unrealistic assumption, for a variety of reasons, including harvesting expenses, as well as industrial use of the crop. In order to release this assumption, Gong and Löfgren (2016) established an age-dependent price function

(13) $$p\left(t\right)=104.63*{\left(t-29\right)}^{0.2602}$$

We will now apply Eq. (13), for *T* > 29, in addition to Eq. (12), in order to establish another version of the practical forestry example.

**Fig. 7:**
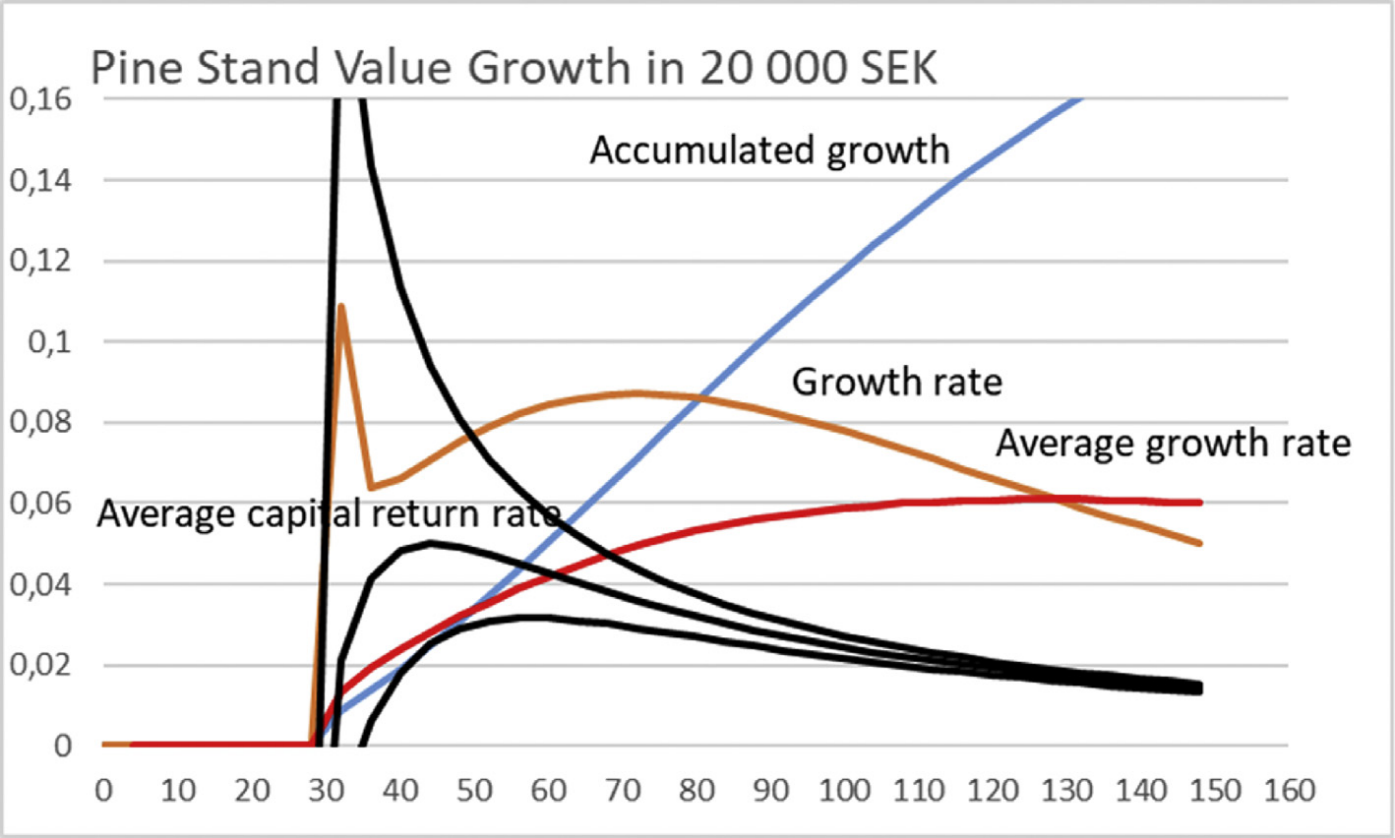
Pine stand value growth according to a North-Swedish value growth function (13), (Gong and Löfgren, 2016). Solid black lines correspond to average financial return rate according to Eq. (3), for three different levels of initial investment. Value growth is given in units of 20 000 SEK, whereas the accumulated growth in millions per hectare.

Fig. 7 shows the outcome for [UO]100=0. Maximum sustainable yield is gained at 130 years of rotation. Applying Eq. (11) provides a capital return of 5.14% at rotation time 44 years, in the absence of any non-operative capitalization.

If the growth could be initiated with a tiny investment of 600 SEK, maximum capital return of 35.2% would be achieved at 30 years of rotation. In case the initial investment would have to doubled to 12 000 SEK, maximum capital return of 3.22% would be achieved at 58 years of rotation.

The above results do not depend on any arbitrary external interest rate. A standard (Faustmann) discounting procedure could be applied for the case. 2% discount interest would yield an optimal rotation time 78 years. A 3% discount interest would yield an optimal rotation of 63 years. For this yield curve, there is not any positive Net Present Value for 4% discount interest. The least amount of money would lost at 60 years of rotation.

**Fig. 8:**
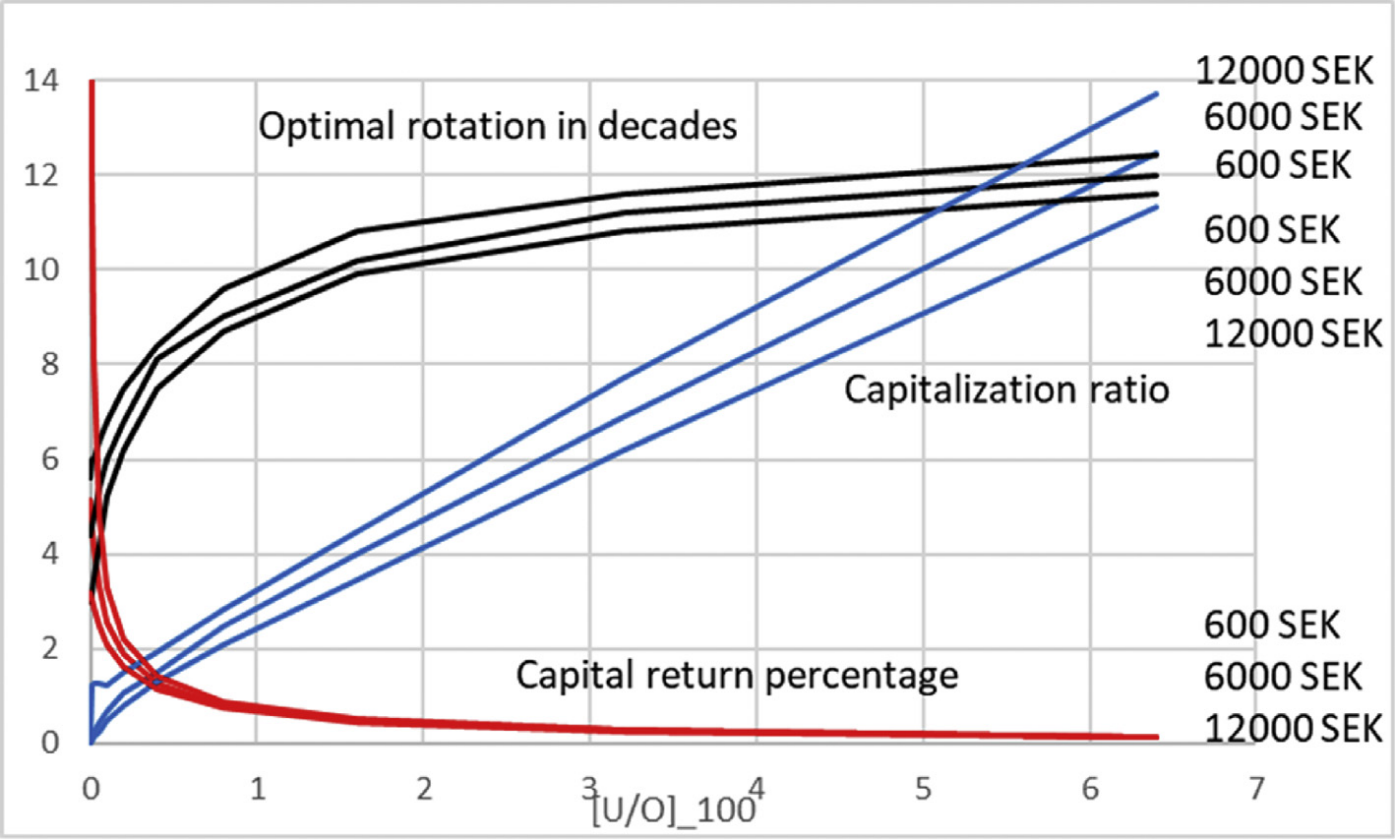
Optimal rotation time, average capitalization ratio 〈U〉〈O〉, and capital return percentage as a function of [UO]100 for three different levels of initial investment for dU/dt=0, using price function (13).

In Fig. 8 we introduce a nonzero non-operative capitalization, but retain dU/dt=0. We plot the optimal rotation time, average capitalization ratio 〈U〉〈O〉 at optimal rotation, as well as the capital return percentage as a function of fixed [UO]100. [UO]100=0 on the left naturally corresponds to the situation illustrated in Fig. 7. Then, as a function of increasing non-operative capitalization, the optimal rotation time evolves towards 130 years, corresponding to greatest possible average net yield rate ΔΩ(τ)/τ. Simultaneously, the capital return rate becomes reduced, and the average capitalization ratio at optimum rotation time 〈U〉〈O〉 increases. Results are plotted for three different values of initial investment, similarly to Fig. 7, and they differ the most at small non-operative capitalization.

**Fig. 9:**
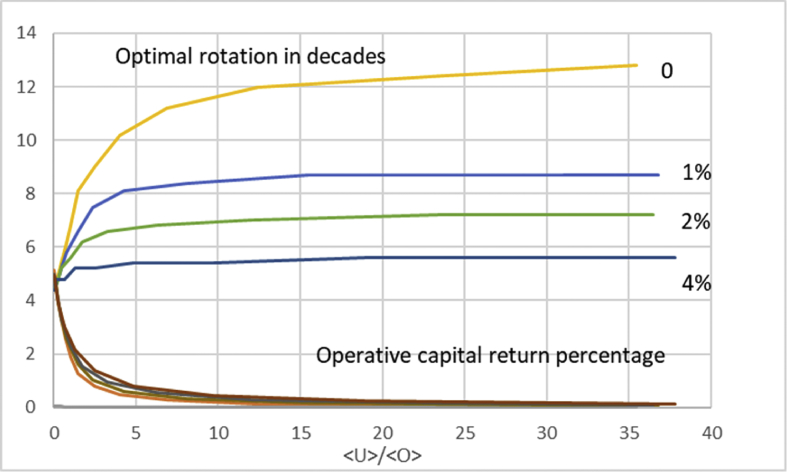
Optimal rotation time and operative capital return percentage as a function of average capitalization ratio 〈U〉〈O〉 at optimum rotation, for four different levels of annual appreciation of non-operative capitalization, using price function (13).

In Fig. 9 we retain a variety of values of non-operative capitalization, but introduce a nonzero change rate dU/dt. There is only one value of initial investment, 6000 SEK/ha. We arrange the data according to average capitalization 〈U〉〈O〉 at the instant of maximum operative capital return $\frac{\Delta \Omega \left(\tau \right)}{\tau \left[\langle O\rangle +\langle U\rangle \right]}$ (cf. Eq. (11)). Interestingly, the maximal operative capital return is almost independent on the appreciation rate of the non-operative capitalization. Still more interestingly, the optimal rotation time is rather sensitive to the appreciation rate. Perhaps most interestingly, the optimal rotation age is a rather weak function of the level of non-operative capitalization; in the case of 4% appreciation, increasing non-operative capitalization does not increase the optimal rotation time beyond 56 years.

**Fig. 10:**
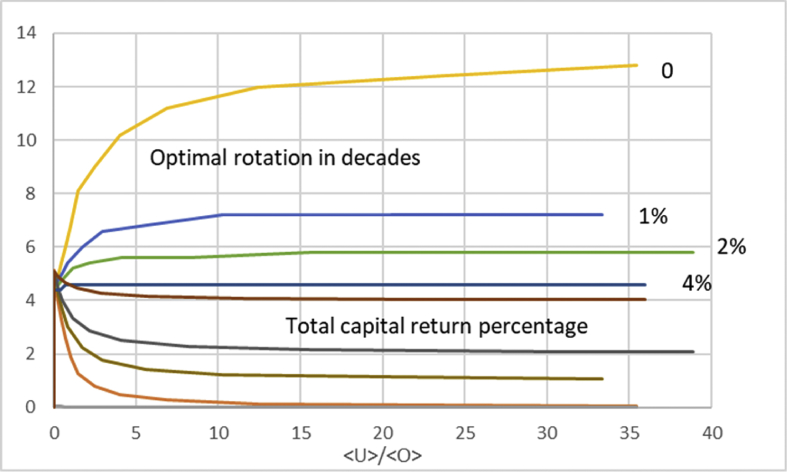
Optimal rotation time and total capital return percentage as a function of average capitalization ratio 〈U〉〈O〉 at optimum rotation, for four different levels of annual appreciation of non-operative capitalization, using price function (13).

In Fig. 10 we discuss total return of capital, instead of the operative return. We again arrange the data according to average capitalization 〈U〉〈O〉, but now at the instant of maximum total capital return $\frac{\Delta \Omega \left(\tau \right)+\Delta U\left(\tau \right)}{\tau \left[\langle O\rangle +\langle U\rangle \right]}$ (cf. Eq. (3)). It is found that the maximal total capital return approaches the appreciation rate of the non-operative capitalization along with increasing non-operative capitalization. More interestingly, the non-operative appreciation rate strongly contributes to the effect of non-operative capitalization on the optimal rotation time. At zero appreciation, the optimal rotation time approaches the Maximum Sustainable Yield rotation along with non-operative capitalization, as already recognized from Eq. (11) and from Figs. 8 and 9. However in the case of appreciating non-operative capitalization this does not happen. As a function of increasing non-operative capitalization, the optimal rotation time approaches a constant that rather significantly differs from the MSY-rotation. In the case of 4% annual non-operative appreciation, the optimal rotation time only increases to 46 years, from the optimum of 44 at zero non-operative capitalization.

**Fig. 11:**
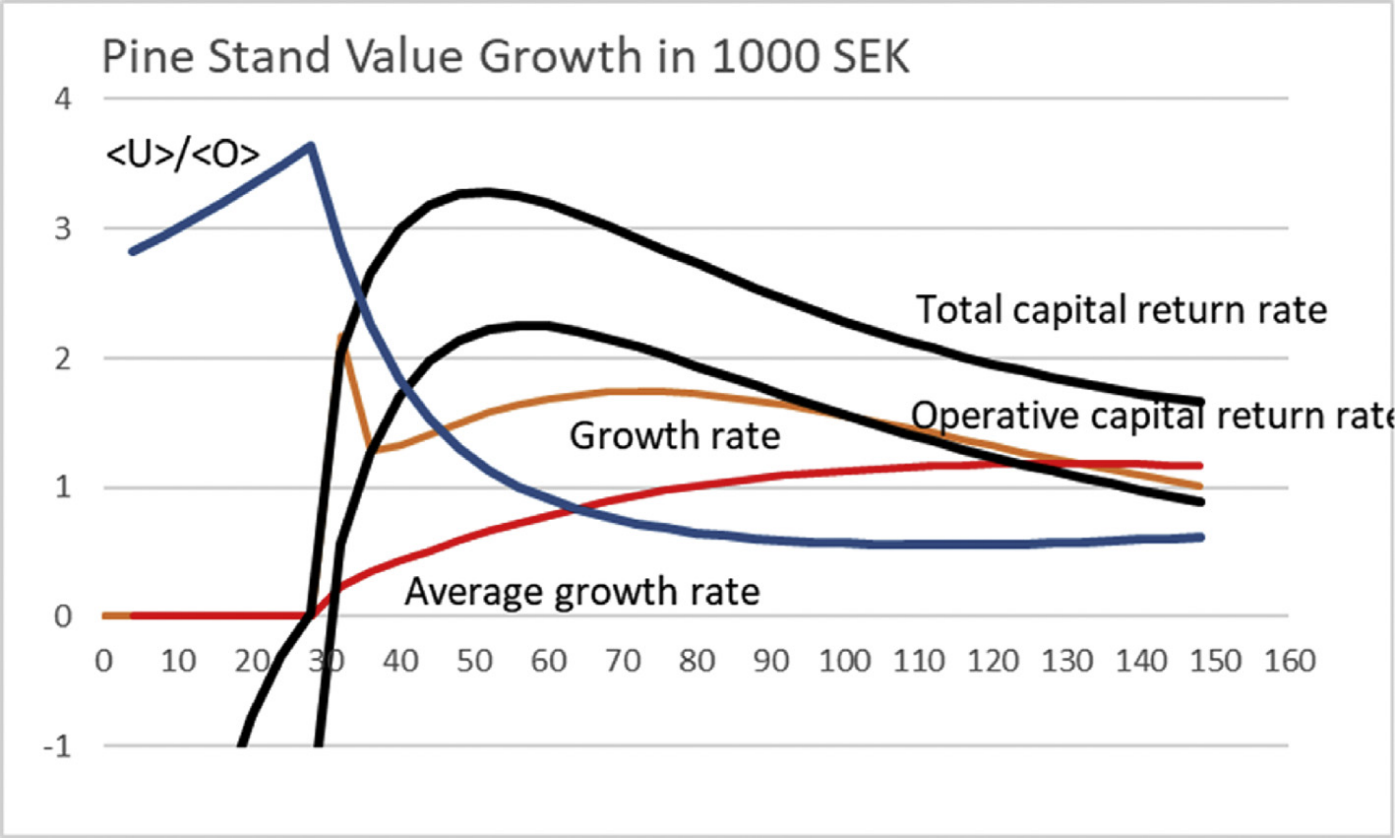
Total and operative capital return percentages as a function of rotation time for Pine stand growth functions (12) and (13). The Figure also displays average capitalization ratio 〈U〉〈O〉. Non-operative capital appreciation rate is 2%, [UO]100=0.5.

Deeper understanding of the results shown in Figs. 9 and 10 probably requires discussion of capital return as a function of rotation time in the presence on non-operative capitalization. We show the operative and the total capital return as a function of rotation time in Fig. 11, where [UO]100=0.5, and non-operative capital appreciation rate is 2%. It is found that the maximum total capital return rate is found at rotation time 52 years, where the average capitalization ratio is 1.16. The maximum operative capital return rate is found at rotation time 56 years, where the average capitalization ratio is 1.03. Beyond 120 years of rotation, the total capital return rate is below the non-operative capital appreciation rate.

**Fig. 12:**
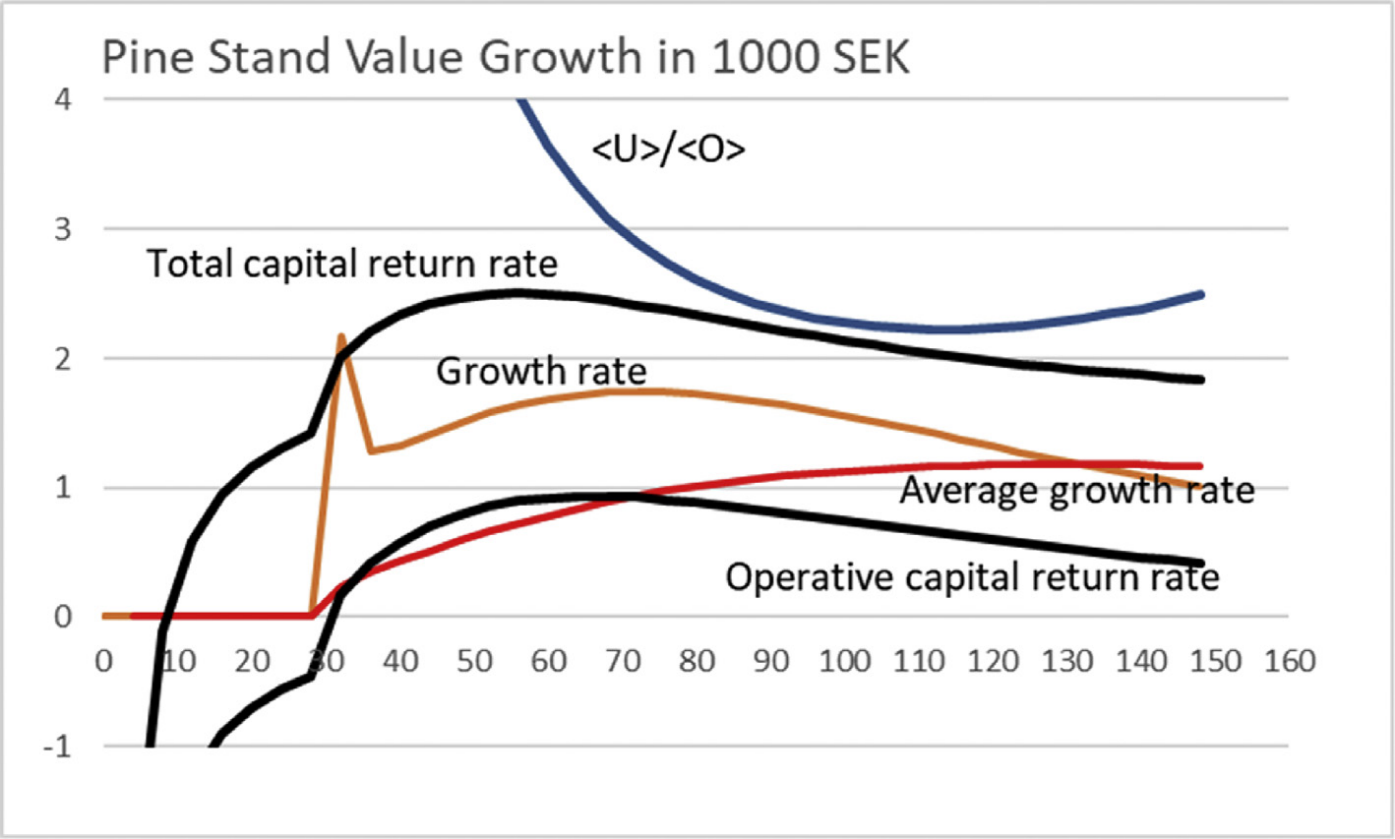
Total and operative capital return percentages as a function of rotation time for Pine stand growth functions (12) and (13). The Figure also displays average capitalization ratio 〈U〉〈O〉. Non-operative capital appreciation rate is 2%, [UO]100=2.

One can, again, recognize that the situation depicted in Fig. 11 is not stationary in the long term. In case the non-operative capitalization keeps appreciating, the boundary condition [UO]100 evolves. With 2% appreciation rate of the non-operative capitalization, after 35 years [UO]100=1. After another 35 years, [UO]100=2. Fig. 12 shows the capital return rates, as well as the capitalization ratio, for the latter value of the boundary condition. It is found that the maximum total capital return rate is found at rotation time 56 years, where the average capitalization ratio is 4.11 (value does not appear the Figure). The maximum operative capital return rate is found at rotation time 66 years, where the average capitalization ratio is 3.24. Beyond 120 years of rotation, the total capital return rate is below the non-operative capital appreciation rate. It is worth noting that even if [UO]100=2, [〈U〉〈O〉]100>2.

### 3.3 Long-term solutions

It is found from Figs. 3, 4, 5, 6 and 9, 10, 11, 12 that appreciating non-operative capitalization leads to rather low operative capitalizations, in order to maximize capital return within a particular growing cycle. However, Eqs. (1), (2) and (3) show that along with appreciating non-operative capitalization, the operative capital return approaches zero, and the total capital return the appreciation rate of the non-operative capitalization. In such circumstances, other forms of capital use may become appropriate, instead of growing crops without gaining any operative return of it. However it is of interest how a long-term appreciation of the non-operative capitalization would affect financial sustainability, provided multiannual plants are continuously cultivated.

Firstly, let us denote the total capital return within growth cycle *i*, according to Eq. (11), as

(14) $$T_{i}=\frac{\Delta \Omega_{i}+\Delta U_{i}}{\tau_{i}{\langle K\rangle }_{i}}$$

The expected value of total capital return over a longer period is, in analogy with Eq. (11)

(15) $$\langle T\rangle =\frac{1}{\langle \langle K\rangle \rangle }\frac{\sum \tau_{i}T_{i}{\langle K\rangle }_{i}}{\sum \tau_{i}}$$

where 〈〈K〉〉 is the grand mean of capitalization over time.

Provided the applied rotation $\tau_{i}$ is independent of *i*,

(16) $$\langle T\rangle =\frac{1}{\langle \langle K\rangle \rangle }\frac{\sum T_{i}{\langle K\rangle }_{i}}{n}$$

where *n* is the number of growth cycles. Then, Eq. (16) can be rewritten

(17) $$\langle T\rangle =\frac{1}{n\tau \langle \langle K\rangle \rangle }\sum \left(\Delta \Omega_{i}+\Delta U_{i}\right)=\frac{1}{n\tau \left[\langle O\rangle +\langle \langle U\rangle \rangle \right]}\sum \left(\Delta \Omega_{i}+\Delta U_{i}\right)$$

Inserting appropriate expressions for the sum and the mean, one can readily rewrite Eq. (17) as

(18) $$\langle T\rangle =\frac{1}{\tau }\frac{\Delta \Omega +\frac{U_{0}}{n}\left(e^{un\tau }-1\right)}{\langle O\rangle +U_{0}\frac{e^{un\tau }-1}{un\tau }}$$

where *u* is the non-operative capitalization appreciation rate.

Taking the high-capitalization boundary condition 〈O〉<<〈〈U〉〉 results as

(19) $${\langle T\rangle }_{\langle O\rangle <<\langle \langle U\rangle \rangle }=\frac{nu\Delta \Omega }{U_{0}\left(e^{un\tau }-1\right)}+u$$

**Fig. 13:**
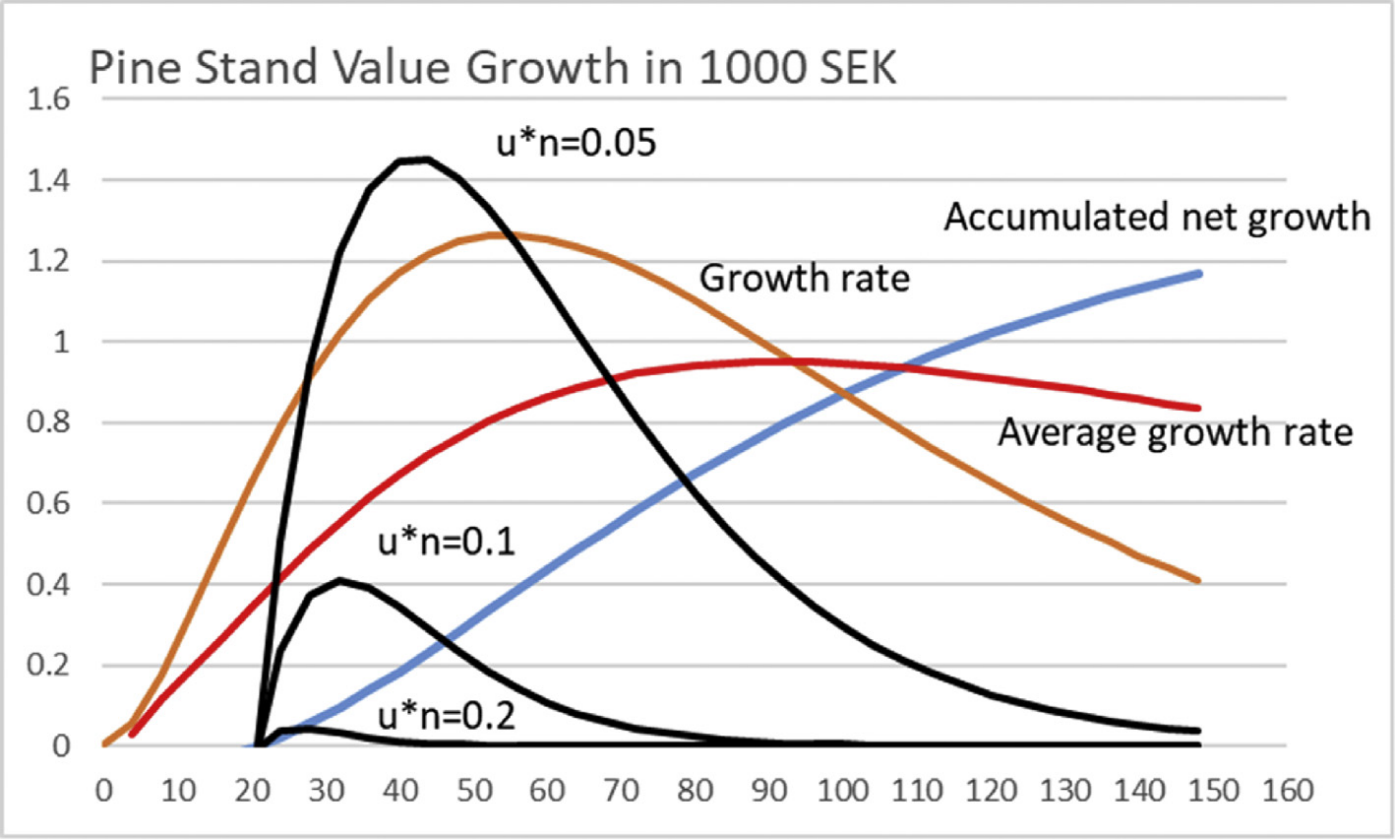
The factor $\frac{n*u*\Delta \Omega }{\left(e^{un\tau }-1\right)}$ from Eq. (19), magnified for clarity, for three different combinations rates u*n, as a function of rotation time *τ*, for Pine stand growth function (12). The accumulated net growth appearing in Eq. (19) is in 100 000 SEK, the growth rate and average growth rate in 1000 SEK/year.

The first term of Eq. (15) naturally refers to operative capital return rate, whereas the second to non-operative capital return rate. We readily find that the operative capital return vanishes with increasing *unτ*. However, there is a particular optimum range for the rotation time *τ* for any combination of appreciation rate *u* and number of growth cycles *n*. The combination $\frac{nu\Delta \Omega }{\left(e^{un\tau }-1\right)}$ is plotted or a variety of values of *un* in Fig. 13, using the growth function appearing in Eq. (12). It is found that for *un* = 0.05, the optimal rotation time *a* corresponds to 42 years. Increasing the values of *un* to 0.1 and 0.2, reduces the rotation time to 32 and 26 years, respectively. One can readily verify that as *un* approaches zero, the solution approaches the rotation of 95 years, corresponding to maximal sustainable yield.

**Fig. 14:**
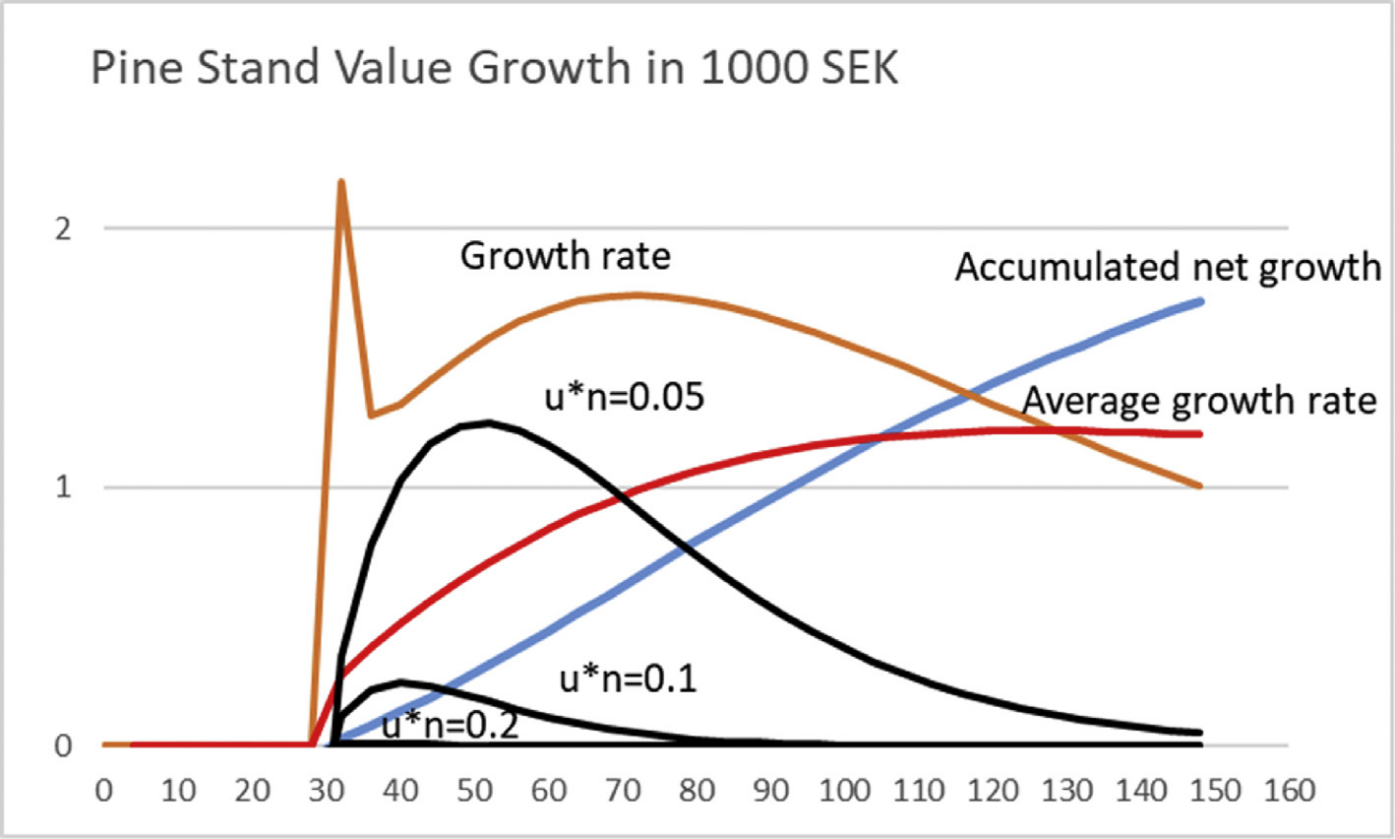
The factor $\frac{n*u*\Delta \Omega }{\left(e^{un\tau }-1\right)}$ from Eq. (19), magnified for clarity, for three different non-operative appreciation rates u*n, as a function of rotation time *τ*, for Pine stand growth function (12). The accumulated net growth appearing in Eq. (19) is in 100 000 SEK, the growth rate and average growth rate in 1000 SEK/year. Price function (13) is used.

Using the price function (13) in addition to the growth function (12), it is found that the rotation corresponding to the maximal long-term capital return according to Eq. (19) again significantly depends on the appreciation rate of the non-operative capitalization, multiplied by the number of cycles *un*. We find from Fig. 14 that for *un* = 0.05, the optimal rotation time *τ* corresponds to 50 years. Increasing the values of *un* to 0.1 and 0.2, reduces the rotation time to 40 and 34 years, respectively. One can readily verify that as *un* approaches zero, the solution approaches the rotation of 130 years, corresponding to maximal sustainable yield.

### 3.4 Intermediate capitalizations

Figs. 13 and 14 being drawn with the boundary condition 〈O〉<<〈〈U〉〉, and indicating financial sustainability of rather low operative capitalizations, it is of interest to consider what kind of operative capitalizations would be feasible for repeated growth cycles at intermediate capitalizations. In the case of growth function (12), this can be readily done by discussing Figs. 5 and 6. Fig. 5 corresponding to [UO]100=1 and *u* = 2%, shows optimal rotations of 46 and 52 years, in relation to total and operative return, respectively. On the other hand, in Fig. 6 the first boundary condition is increased to [UO]100=4, resulting as optimal rotations 49 and 56 years. We conclude that along with further rotation cycles, the result changes only slightly. This result is in concert with Figs. 3 and 4, which also indicate that the increment of the optimal rotation ceases with further capitalization. The rotations do not differ much from corresponding long-term solutions in Fig. 13. It appears that non-operative capitalization appreciation rate greater than 2% pushes for still shorter rotations (Figs. 3 and 4). Long rotation times, approaching the maximum sustainable yield, are financially sustainable only in the case of high capitalization and negligible non-operative appreciation rate combined (Eq. (11), Figs. 2, 3, and 4). In the mind of the author, such a case is a curiosity, probably not frequently appearing in real life.

In the case of applying the value function (13) in addition to the growth function (12), one can consider what kind of operative capitalizations would be feasible for repeated growth cycles at intermediate capitalizations by discussing Figs. 11 and 12. Fig. 11 corresponding to [UO]100=0.5 and *u* = 2%, shows optimal rotations of 52 and 56 years, in relation to total and operative return, respectively. On the other hand, in Fig. 12 the first boundary condition is increased to [UO]100=2, resulting as optimal rotations 56 and 66 years. We conclude again that along with further rotation cycles, the result changes only moderately. This result is in concert with Figs. 9 and 10, which also indicate that the increment of the optimal rotation ceases with further capitalization. The rotations do not differ much from corresponding long-term solutions in Fig. 14. It appears that non-operative capitalization appreciation rate greater than 2% pushes for still shorter rotations (Figs. 9 and 10). Long rotation times, approaching the maximum sustainable yield, are financially sustainable only in the case of high capitalization and negligible non-operative appreciation rate combined (Eq. (3), Figs. 8, 9, and 10). Such a case, again, probably does not frequently appear in real life.

## 4 Discussion

We have introduced a momentary capital return rate function, the expected value of capital return rate, as well as the expected value of capital return rate in time-age domain. The capital return rate depends on any yield function, or value growth function, describing the increment rate of capitalization. Provided the value growth function can be properly established for local circumstances, the introduced return rate function can be used for the design of technical operations and commercial transactions. The financial treatment is not necessarily limited to forest trees but may be applicable to other multiannual plants, like bamboo, provided appropriate yield functions are available (Wi et al., 2017). Further, the methods are not necessarily limited to biological growth processes – whatever growing business can be treated similarly, provided the yield function can be approximated.

The approach introduced in this paper is obviously new, at least within the business of periodically growing multiannual plants like forest trees. The momentary capital return rate in Eq. (1) is related to the Pressler's indicator rate (1860), which however has not been widely applied within the field. The equality to Pressler applies only under the boundary condition of invariable non-operative capitalization, which according to the results of this paper would be a rather strong assumption. Further, Pressler's indicator rate corresponds to momentary capital return, not to the relevant expected values appearing in Eqs. (2), (5), (10), and (11).

The momentary capital return rate, as well as the expected value over a rotation, can be applied to any individual stand. We have succeeded showing that the expected value is the same as the capital return rate for a forest estate with evenly distributed stand ages. In other words, Eqs. (5) and (10) are the same, provided stand ages are evenly distributed.

It appears that the appreciation rate of non-operative capitalization is a factor dominating sustainable management practices. The range of the annual appreciation rate investigated was from zero to 4%. The validity of this range was verified using trade statistics of forest estates in Finland, from 1990 to 2018.

Most of the numerical results in this paper have been discussing optimal rotation times. This is because the rotation times can easily be determined on the basis of the yield function (12) and the value function (13), and they do constitute easily comprehensible example applications. The results however can be easily compared with those of a recent study regarding continuous-cover forestry with frequent diameter-limit cutting (Kärenlampi, 2018). In both cases, low non-operative capitalization favors low operative capitalization, which in turn corresponds to either young rotation age or low cutting limit diameter. High but stationary non-operative capitalization favors high operative capitalization, even up to that corresponding to maximum sustainable yield. High increment rate of non-operative capitalization favors low operative capitalization, even if the magnitude of the non-operative capitalization would be high (Figs. 4 and 10, Kärenlampi, 2018).

There are many operations which can be designed on the basis of capital return, including soil preparation, planting or seeding, young stand cleaning, precommercial thinning, fertilization, drainage, and commercial thinning. Commercial thinning cycles may be designed to harvest either small or large trees, or a combination of both. All these activities have an immediate effect on the capitalization, as well as a non-immediate effect on growth and correspondingly future capitalization.

In the present paper, no external-interest discounting is applied, and neither are any cash flows emphasized. We feel that in the economical environment of the 21^st^ century, financial considerations are better justified, in comparison to cash flows. Wealth predominantly appears as codes and numbers within information systems, and can be liquidized within seconds. Consequently, capital appreciation rate, instead of cash flow, has become a feature dominating economic activity. In addition, it has been recently shown that application of external interest rates may have devastating financial consequences (Kärenlampi, 2019a).

## Declarations

### Author contribution statement

Petri P. Kärenlampi: Conceived and designed the analysis; Analyzed and interpreted the data; Contributed analysis tools or data; Wrote the paper.

### Funding statement

This research did not receive any specific grant from funding agencies in the public, commercial, or not-for-profit sectors.

### Competing interest statement

The authors declare no conflict of interest.

### Additional information

No additional information is available for this paper.
